# Development of a CT-based comprehensive model with deep learning for differentiating pathological types of pulmonary ground-glass nodules

**DOI:** 10.3389/fmed.2026.1831127

**Published:** 2026-05-26

**Authors:** Jian Zhang, Boheng Liu, Ji Li, Yang Liu, Jipeng Jiang

**Affiliations:** 1 School of Medicine, Nankai University, Tianjin, China; 2 Department of Thoracic Surgery, The First Medical Center of Chinese PLA General Hospital, Beijing, China; 3 Postgraduate School, Medical School of Chinese PLA, Beijing, China; 4 Department of Radiation Oncology, Shandong Cancer Hospital and Institute Affiliated to Shandong First Medical University, Shandong Academy of Medical Sciences, Jinan, Shandong, China

**Keywords:** deep learning, ground-glass opacities, machine learning, pulmonary pathology, radiomics

## Abstract

**Background:**

The lack of reliable clinical features for differentiating benign from malignant pulmonary pure ground-glass nodules (pGGNS) leads to potential misdiagnosis and unnecessary invasive examinations. Although radiomics and deep learning approaches have shown potential in nodule characterization, the diagnostic performance of integrated models combining clinical features, radiomics, and deep learning remains insufficiently defined. This study aimed to develop and validate an integrated model to distinguish benign from malignant pGGNs and to further differentiate pathological subtypes.

**Materials and methods:**

This retrospective study included 1,067 patients with pulmonary pGGNs from Shandong First Medical University Cancer Hospital. Clinical and imaging data were collected, and radiomics features and deep learning (DL) derived features were extracted using Python (version 3.7). Patients were randomly divided into training and validation cohorts. Multiple machine-learning classifiers were constructed, and diagnostic performance was assessed using receiver operating characteristic (ROC) curve analysis.

**Results:**

For distinguishing benign from malignant pGGNS (Model 1), clinical features such as age, nodule multiplicity, CEA levels, and amylase were identified as clinically relevant features. Thirty-eight valuable features were selected for model development. Among individual classifiers, the Support Vector Machine (SVM) achieved the highest performance with a validation receiver operating characteristic curve (AUC) of 0.840, followed by random forest (0.829), stochastic gradient descent (0.828), k-nearest neighbors (0.814), XGBoost (0.798), and LightGBM (0.818). The integrated model combining clinical features, radiomics, and deep learning achieved a validation set AUC of 0.871. For pathological subtype classification of pGGNs (Model II), clinical features such as gender, Pro-Gastrin-Releasing-Peptide (ProGRP), AST/ALT ratio (De Ritis ratio), creatine kinase-MB (CKMB), and globulin were identified as informative clinical variables. Twelve valuable features were selected The SVM classifier again showed the best individual performance (validation AUC = 0.831), while the integrated model achieved a superior AUC of 0.853.

**Conclusion:**

An integrated model incorporating clinical characteristics, radiomics, and deep learning demonstrates robust performance in distinguishing benign from malignant pulmonary pGGNs and in identifying pathological subtypes, suggesting potential clinical utility for non-invasive decision support.

## Introduction

Cancer remains a leading cause of death worldwide (1, 2). The Cancer Atlas reveals approximately 23.6 million (95% UI: 22.2–24.9 million) new cancer cases and 10 million (95% UI: 9.36–10.6 million) cancer-related deaths occurred globally in 2019 (3). Lung cancer represents the most prevalent tumor and the leading cause of cancer-related mortality worldwide, largely attributable to smoking and environmental pollution (4). In 2022, an estimated 4.824 million new cases and 3.21 million cancer-related deaths occurred in China, compared to 2.37 million new cases and approximately 640,000 deaths in the United States. Although the age-standardized incidence rate (ASIR) in the United States (303.6 per 100,000) was significantly higher than that in China (201.61 per 100,000), the age-standardized mortality rate (ASMR) in China (96.47 per 100,000) exceeded that of the United States (81.8 per 100,000), reflecting disparities in cancer profiles, healthcare infrastructure, and early detection strategies between the two countries (5).

Early diagnosis and timely intervention are particularly critical for lung cancers presenting as ground-glass nodules (GGNs), as prognosis is closely related to disease stage at detection (6, 7). Data from the International Early Lung Cancer Action Plan reported that early detection and surgical resection of lung cancer presenting as GGNs or partially solid nodules could achieve near-perfect lung cancer-specific survival (6, 7).

The widespread adoption of low-dose computed tomography (LDCT) has significantly increased the detection rate of pulmonary nodules (8). The National Lung Screening Trial (NLST) reported a 20% reduction in lung cancer mortality with LDCT screening compared with chest radiography (8). However, distinguishing benign from malignant GGNs remains challenging in clinical practice. Pure ground-glass nodules (pGGNs) exhibit limited specificity on conventional CT imaging, and their morphological features often overlap between benign and malignant entities (9, 10). pGGNs may indicate benign conditions such as inflammation, hemorrhage, edema, and focal fibrosis, or they may suggest malignant tumors. Studies indicate that 63–92.6% of persistent pGGNs represent precancerous lesions or early adenocarcinoma (9, 10).

Despite advances in imaging technology, pGGNs may still be misdiagnosed due to factors including small size, indistinct margins, inconspicuous appearance, or proximity to vascular structures (11). Consequently, a considerable proportion of patients undergo unnecessary invasive diagnostic procedures or surgical interventions, underscoring the need for accurate, non-invasive methods to stratify malignant risk in pGGNs.

Radiomics (RAD) enables the high-throughput extraction of quantitative features from medical images using advanced mathematical algorithms and machine-learning techniques, providing objective descriptors of lesion heterogeneity beyond visual assessment (12). As radiomics research deepens, it is applied not only to the overall presentation of lesions but also to disease diagnosis, staging, prognosis, and evaluation of treatment efficacy (12). The advantage of radiomics lies in its ability to reveal clinical outcomes and guide clinical decisions through non-invasive means, offering new pathways for disease diagnosis and treatment. Deep learning (DL), with its hierarchical neural network architecture, further enhances feature learning capacity and scalability in large imaging datasets (13). The integration of DL with radiomics has shown promise across multiple medical domains (14, 15). However, evidence remains limited regarding the application of integrated clinical, radiomics, and DL models for differentiating pathological subtypes of pulmonary pure ground-glass nodules.

Accordingly, this study aimed to develop and validate multimodal machine learning models for the non-invasive characterization of pulmonary pGGNs. First, we constructed an integrated model combining clinical variables, radiomics, and deep learning features to distinguish malignant from benign pGGNs and to estimate malignant risk. Furthermore, we developed a subtype classification model to differentiate pGGNs with malignant potential (including atypical adenomatous hyperplasia and adenocarcinoma *in situ*) from pGGNs without malignant potential, with the goal of supporting individualized treatment decision-making.

## Materials and methods

### Patient population

Retrospective collection of patients with pulmonary pure ground-glass nodules (pGGNs) who underwent surgical treatment at Shandong First Medical University Affiliated Cancer Hospital from August 2023 to August 2025.

### Inclusion criteria

1. Complete clinical, pathological, and imaging data are available.
2. Follow-up was performed at 1 or 3 months prior to surgery.
3. The nodule without any solid components.
4. Surgical treatment was performed, with definitive pathological findings obtained postoperatively.

### Exclusion criteria

1. pGGNs following radiotherapy or chemotherapy.
2. pGGNs after anti-inflammatory treatment.
3. Invasive procedures before surgery, such as nodule biopsy or radiofrequency ablation.
4. History of other concurrent malignancies.
5. Poor image quality due to respiratory movement or other artifacts.

This study was approved by the Ethics Committee of Shandong First Medical University Affiliated Cancer Hospital (Approval No. SDTHEC202509033).

### Clinical features assessment

The clinical variables collected included age, gender, BMI, hypertension, diabetes, smoking history, tumor history, family history, anti-inflammatory therapy, routine laboratory test results and CT scan findings. Laboratory tests encompassed routine blood examinations and common lung cancer tumor markers, such as Pro-Gastrin-Releasing Peptide (ProGRP), Carcinoembryonic Antigen (CEA), Cytokeratin 19 Fragment (CYFRA21-1), Neuron-Specific Enolase (NSE), and Squamous Cell Carcinoma Antigen (SCC).

Conventional semantic CT features were independently evaluated by two radiologists with more than 5 years of experience, blinded to pathological results. Assessed features included lesion location, margin characteristics (smooth or blurred), maximum diameter, proximity to the pleura (yes/no), anatomical location (central or peripheral), and nodule multiplicity (single or multiple). Additional clinical characteristics were retrospectively extracted from electronic medical records at Shandong First Medical University Cancer Hospital (Supplementary Appendices I, II).

### Pathological diagnosis

All pathological diagnoses were based on postoperative surgical specimens. To ensure the reliability of the pathology findings, all patient diagnoses underwent retrospective review by a pathologist with o more than 10 years of experience at two hospitals. All pathology results were confirmed to be reliable.

### Image acquisition and preprocessing

The original medical imaging data were acquired using SIEMENS SOMATOM Definition AS and SIEMENS SOMATOM Definition Flash dual-source CT scanners for chest CT examinations and stored in DICOM format. The SimpleITK tool was employed to batch convert all DICOM data into NIfTI format (.nii.gz). This ensured that the converted data fully preserved the spatial position information and grayscale distribution of the images, while facilitating subsequent processing workflows.

All images were converted and normalized using an open-source Python 3.7 package. These preprocessed images were then imported into the 3D Slicer 5.2.2 software to adjust to uniform grayscale parameters.

Following this preprocessing step, manual segmentation was performed on the 1.5 mm thin-slice images from the preoperative plain CT scans. Normalized image data were loaded using 3D Slicer software (version 5.2.2). Using its “Segment Editor” module, two radiologists manually delineated regions of interest (ROIs) in a randomized order of cases. After delineation of each slice, all segmented slices were fused to generate a volumetric mask.

### Feature extraction and screening in radiomics

Patients were randomly assigned to the training and validation sets at a ratio of 7:3. Radiomics features were extracted from ROIs using an open-source Python-based radiomics library. Based on ROIs, imaging-based shape features, statistical features, and texture features were obtained. The texture feature set was further subdivided into multiple subcategories, including Gray-Level Co-occurrence Matrix (GLCM) features, Gray-Level Run Length Matrix (GLRLM) features, Gray-Level Size-Z-Matrix (GLSZM) features, Neighborhood Gray-Level Difference Matrix (NGTDM) features, and Gray-Level Dependency Matrix (GLDM) features. These multidimensional features comprehensively reflect the geometric morphology, gray-level distribution, and textural heterogeneity of regions of interest, providing rich, in-depth quantitative data support for subsequent machine learning modeling and clinical decision-making.

### Feature stability assessment and radiomics feature construction

The intraclass correlation coefficient (ICC) was used to assess the consistency of features manually delineated by physicians. Features with an ICC > 0.75 were retained. Features in the training set underwent normality tests. Features meeting normality criteria underwent *t*-tests, while non-normally distributed features underwent rank-sum tests for initial screening, with a significance threshold of *P* < 0.05. To avoid multicollinearity, Pearson and Spearman correlation coefficients were used to remove highly correlated features, with a screening threshold of |r| > 0.8. Subsequent refinement employed Lasso regression. The LassoCV method generated 200 candidate lambda values uniformly distributed between 0 and 0.5, with the optimal lambda value selected via 10-fold cross-validation to minimize the mean squared error (MSE). To visually demonstrate the impact of lambda on model performance and coefficients, the program plots the MSE curve against log(Alpha) (including 95% confidence interval error bars) and a path map of Lasso regression coefficients versus lambda, saving these images for future reference. Finally, features with non-zero coefficients corresponding to the optimal lambda are selected, and their names and coefficients are saved. This end-to-end workflow implements data preprocessing, LASSO regression model training, visualization, and result output, providing a foundational data layer for machine learning model development.

### Model development

This study employed six commonly used classifiers for model construction, specifically including Support Vector Machines (SVM), Random Forest, Stochastic Gradient Descent Classifier (SGD), K-Nearest Neighbors (KNN), XGBoost, and LightGBM. To achieve optimal model performance, Bayesian Optimization was utilized for hyperparameter search.

Model validation results are visualized using ROC curves, DCA curves, and calibration curves. To further explore the relationship between radiomic features and model predictions, SHAP (Shapley Additive exPlanations) values were calculated for each feature. The global impact of individual features on model decisions was assessed by analyzing both individual prediction SHAP values and overall feature importance. The primary performance evaluation metrics include accuracy, sensitivity, specificity, F1-score, and AUC. The DeLong test was applied to assess the statistical significance of AUC differences across different groupings within the same machine learning model.

### Statistical analysis

Statistical analysis was performed using SPSS version 23.0 and Python version 3.7. Normally distributed measurement data are presented as mean ± standard deviation ($\bar{x}$ ± s), while non-normally distributed data are expressed as median and interquartile range [M (Q1, Q3)]. Categorical data are reported as counts or percentages.

A comparative analysis was conducted between the training and validation sets, employing Student’s *t*-tests for semantically meaningful quantitative variables and χ^2^ or Fisher’s exact tests for categorical variables. All statistical tests were two-tailed with a significance threshold of *p* < 0.05.

Model performance was assessed using receiver operating characteristic (ROC) curves, incorporating sensitivity, specificity, accuracy (ACC), negative predictive value (NPV), positive predictive value (PPV), and area under the ROC curve (AUC) as evaluation metrics. Calibration curves were used to measure the consistency of observed outcomes. Decision curve analysis (DCA) was employed to determine the clinical utility of the models.

## Result

### Baseline characteristics

A total of 1,067 patients meeting the inclusion criteria were enrolled in this study, including 333 men and 734 women, with a mean age of 55.30 ± 11.34 years (range: 16–81 years). The dataset was divided into training and validation cohorts at an approximate 7:3 ratio. For the benign vs. malignant discrimination model (Model 1, defining the malignant group as positive), the training cohort included 284 malignant and 462 benign cases. In comparison, the validation cohort contained 124 malignant and 197 benign cases. For the model distinguishing benign tumors from atypical and *in situ* carcinomas (Model 2, defining atypical and *in situ* carcinoma as positive), the training cohort comprised 370 positive cases and 91 negative cases, while the validation set contained 163 positive cases and 35 negative cases.

Baseline characteristics were comparable between the training and validation cohorts for both models, with no statistically significant differences observed in clinical variables (all *p* > 0.05), as summarized in Table 1.

**TABLE 1:** Clinical baseline comparison of training and validation sets for model 1 and model 2.

Variable	Model 1			Model 2		
	Training	Validation	*P-*value	Training	Validation	*P-*value
Age	55.00 (49.06, 63.00)	57.00 (48.00, 63.00)	0.725 (M-WU)	53.00 (47.00, 61.00)	54.00 (49.00, 61.00)	0.651 (M-WU)
BMI	23.96 (22.57, 26.67)	24.31 (22.22, 26.45)	0.424 (M-WU)	23.96 (22.44, 26.23)	24.03 (21.94, 26.58)	0.883 (M-WU)
Size (mm)	11.79 (9.00, 16.00)	12.00 (8.00, 16.00)	0.697 (M-WU)	12.00 (9.00, 16.00)	11.79 (9.00, 16.00)	0.582 (M-WU)
Gender			0.179 (χ^2^)			0.999 (χ^2^)
Female	523 (70.1%)	211 (65.7%)		323 (70.1%)	138 (69.7%)	
Male	223 (29.9%)	110 (34.3%)		138 (29.9%)	60 (30.3%)	
Smoking history			0.933 (χ^2^)			1.000 (χ^2^)
No	663 (88.9%)	284 (88.5%)		413 (89.6%)	178 (89.9%)	
Yes	83 (11.1%)	37 (11.5%)		48 (10.4%)	20 (10.1%)	
Tumor history			0.867 (χ^2^)			0.141 (χ^2^)
No	658 (88.2%)	285 (88.8%)		404 (87.6%)	182 (91.9%)	
Yes	88 (11.8%)	36 (11.2%)		57 (12.4%)	16 (8.1%)	
Family history			0.660 (χ^2^)			0.462 (χ^2^)
No	712 (95.4%)	309 (96.3%)		447 (97.0%)	189 (95.5%)	
Yes	34 (4.6%)	12 (3.7%)		14 (3.0%)	9 (4.5%)	
Location			0.012 (χ^2^)			0.828 (χ^2^)
RUL	240 (32.2%)	102 (31.8%)		157 (34.1%)	70 (35.4%)	
LUL	185 (24.8%)	92 (28.7%)		109 (23.6%)	53 (26.8%)	
LLL	172 (23.1%)	51 (15.9%)		99 (21.5%)	36 (18.2%)	
RLL	117 (15.7%)	50 (15.6%)		72 (15.6%)	30 (15.2%)	
RML	32 (4.3%)	26 (8.1%)		24 (5.2%)	9 (4.5%)	
Group			0.917 (χ^2^)			0.611 (χ^2^)
Control	462 (61.9%)	197 (61.4%)		91 (19.7%)	35 (17.7%)	
Experimental	284 (38.1%)	124 (38.6%)		370 (80.3%)	163 (82.3%)	

RUL, Right upper lobe; LUL, Left upper lobe; LLL, Left lower lobe; RLL, Right lower lobe; RML, Right middle lobe; *p-*value for comparing the internal cohort with the external cohort; categorical variables were analyzed by Pearson χ^2^ test and Fisher exact test, continuous variables were compared by Student *t*-test and Mann-Whitney U test.

### Radiomics and clinical signature

In Model 1, multivariable analysis identified age, CEA levels, presence of multiple nodules, and amylase-related parameters as independent predictors for distinguishing malignant from benign pulmonary pure ground-glass nodules (all *p* < 0.05).

**FIGURE 1:**
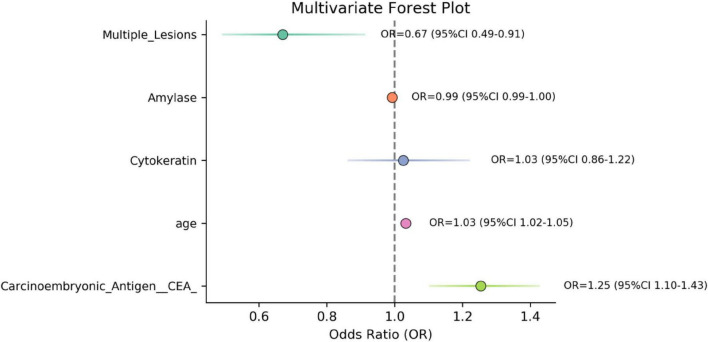
Multifactor analysis forest plot for model 1.

**FIGURE 2:**
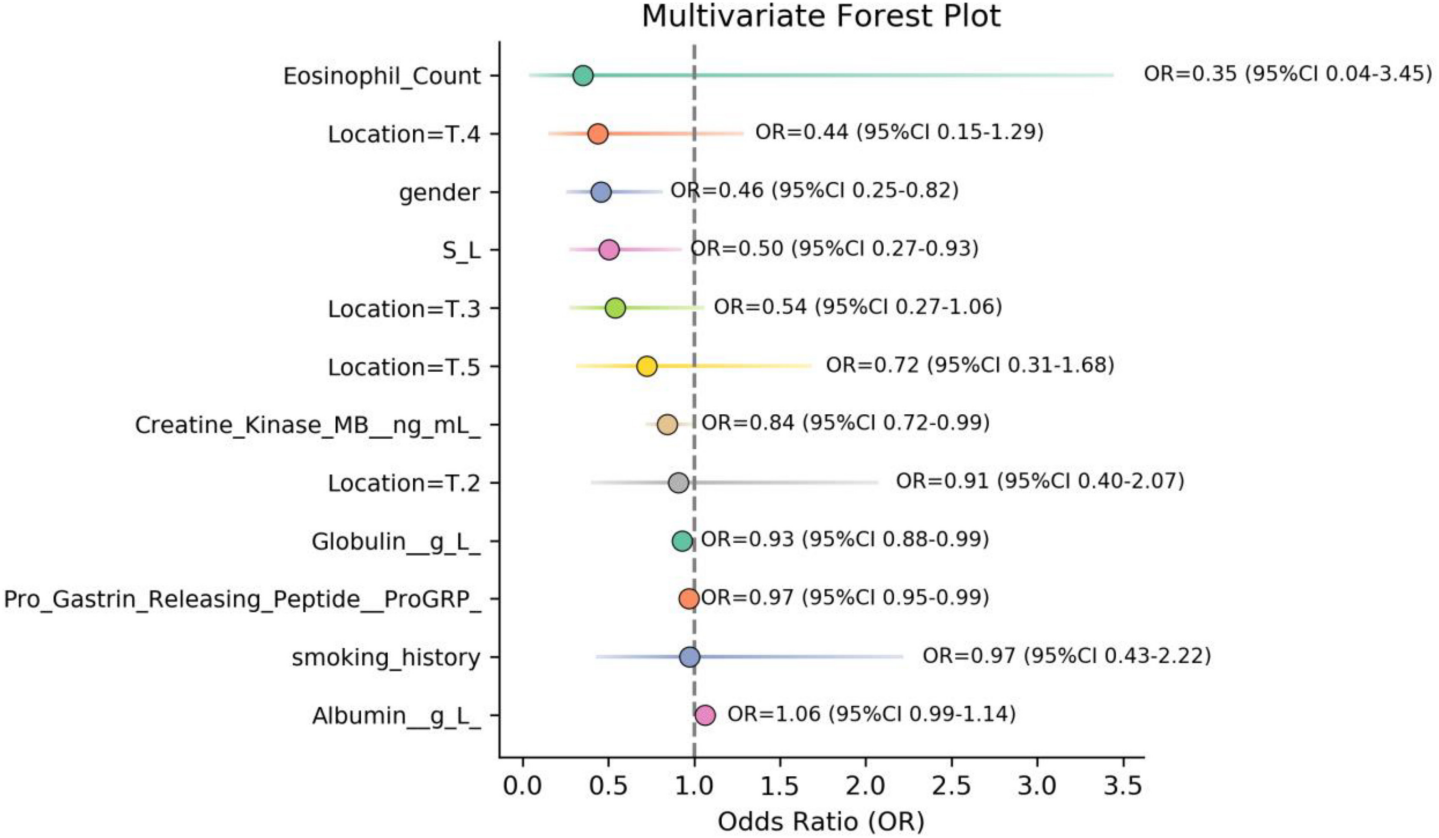
Multifactor analysis forest plot for model 2.

In Model 2, sex, ProGRP, the aspartate aminotransferase to alanine aminotransferase (AST/ALT) ratio (De Ritis ratio), CKMB, and globulin were statistically significant (*p* < 0.05). The multivariate analysis results for Models 1 and 2 are presented in Tables 2, 3, along with Figures 1, 2. Univariate correlation tables are provided in Supplementary Appendices I, II.

**TABLE 2:** Multivariate analysis results for model 1.

Variable	OR	CI95 lower	CI95 upper	*P-*value
Age	1.033	1.016	1.049	0.000
CEA	1.254	1.101	1.427	0.001
Multiple lesions	0.669	0.491	0.913	0.011
Amylase	0.992	0.985	1.000	0.049
Cytokeratin	1.025	0.860	1.222	0.780

CEA, Carcinoembryonic Antigen.

**TABLE 3:** Multivariate analysis results for model 2.

Variable	OR	CI95 lower	CI95 upper	*P-*value
ProGRP	0.969	0.951	0.987	0.001
Gender	0.455	0.253	0.819	0.009
Globulin	0.930	0.875	0.989	0.020
AST/ALT	0.503	0.272	0.929	0.028
Creatine kinase MB	0.843	0.717	0.992	0.040
Albumin	1.062	0.992	1.135	0.082
Eosinophil count	0.353	0.036	3.449	0.370
Smoking history	0.972	0.426	2.219	0.946

ProGRP, Pro Gastrin Releasing Peptide; AST/ALT, Aspartate aminotransferase to alanine aminotransferase.

### Feature selection results

In the training cohorts for Models 1 and 2, features with an intraclass correlation coefficient (ICC) > 0.75 were selected for future analysis. Subsequently, normality tests were conducted on these features, followed by preliminary significance analysis using *t*-tests or rank-sum tests, as appropriate. Highly correlated features (|r| > 0.8) were then eliminated using Pearson’s or Spearman’s correlation coefficients. Finally, LASSO regression was applied, resulting in the selection on 38 features for Model 1 and 12 features for Model 2.

**FIGURE 3:**
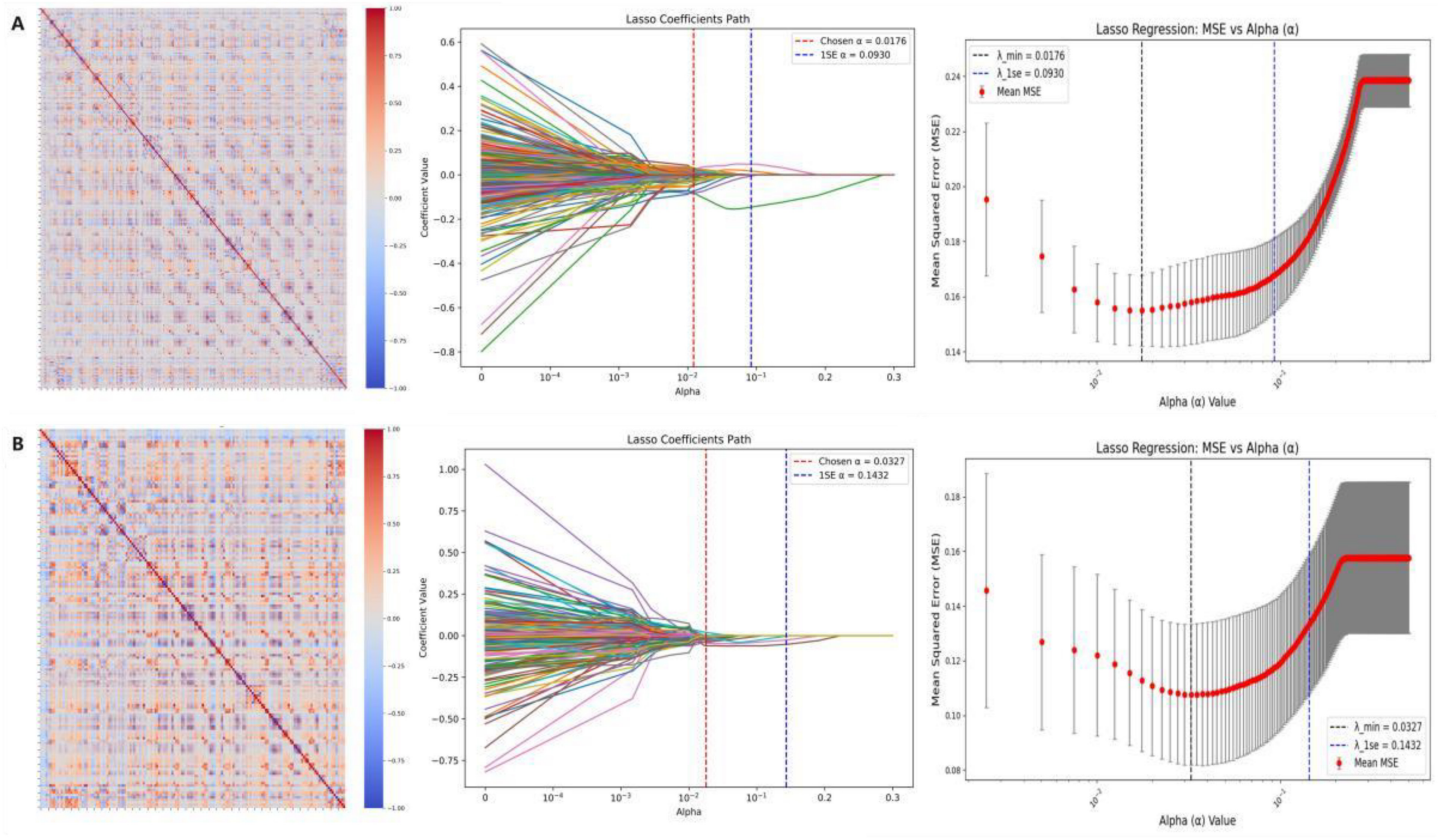
Correlation analysis heatmap and LASSO regression plot for model 1 **(A)** and model 2 **(B)**.

**FIGURE 4:**
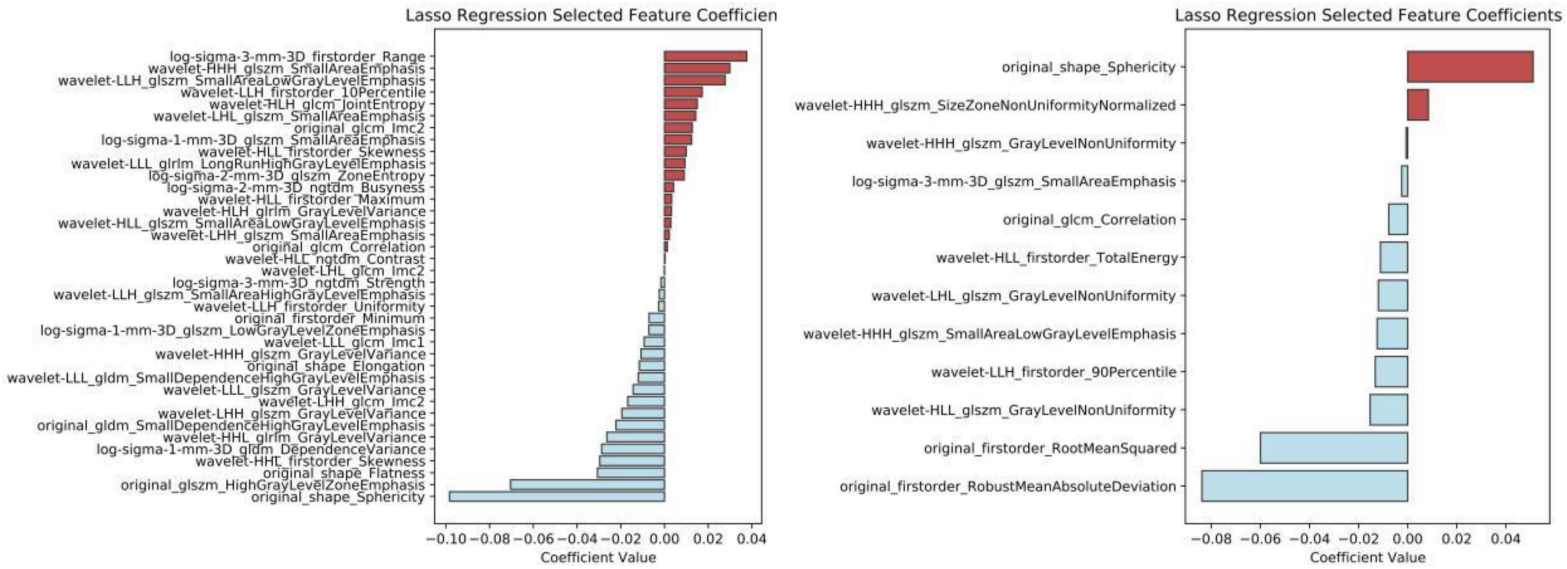
Bar charts of retained features and coefficients following LASSO regression screening for Model 1 (left) and Model 2 (right).

The feature correlation heatmap and LASSO regression workflow are presented in Figure 3. The retained features along with their respective weights in the LASSO regression are shown in Figure 4.

### Model performance

**FIGURE 5:**
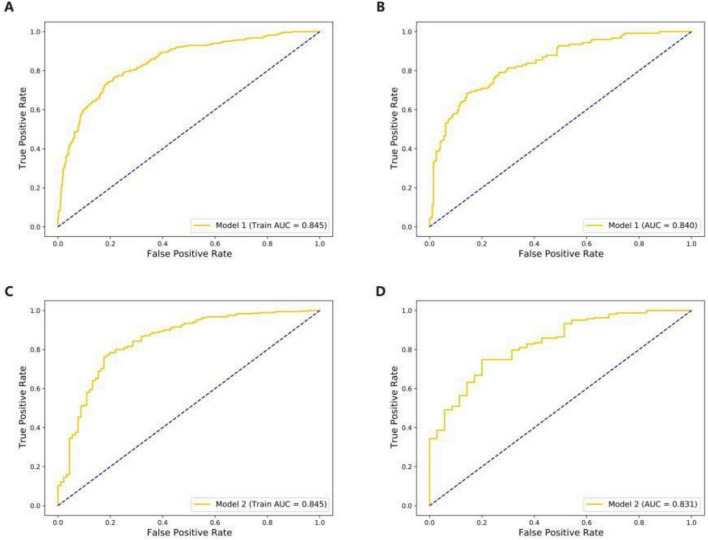
ROC curves for the training and validation sets under the SVM machine learning classifier. **(A)** Model 1 training set, **(B)** model 1 validation set, **(C)** model 2 training set, **(D)** model 2 validation set.

**FIGURE 6:**
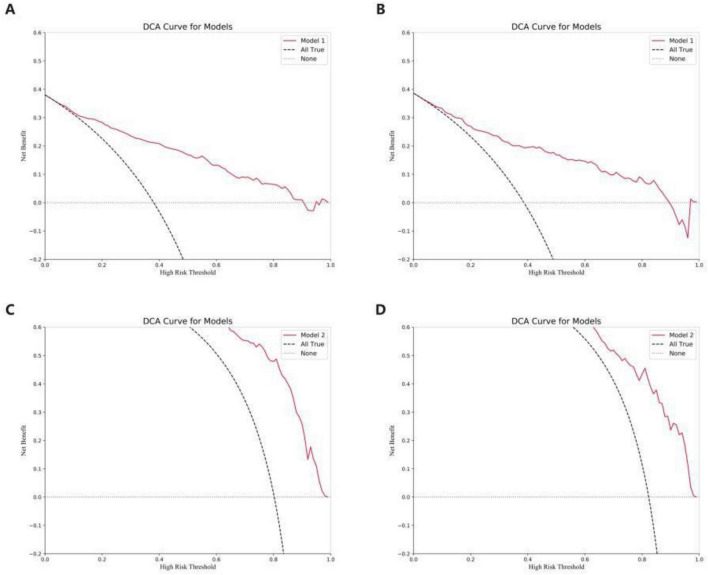
Decision curve analysis (DCA) curves for the training set **(A)** and validation set **(B)** under the SVM machine learning classifier. **(A)** Model 1 training set, **(B)** model 1 validation set, **(C)** model 2 training set, **(D)** model 2 validation set.

**FIGURE 7:**
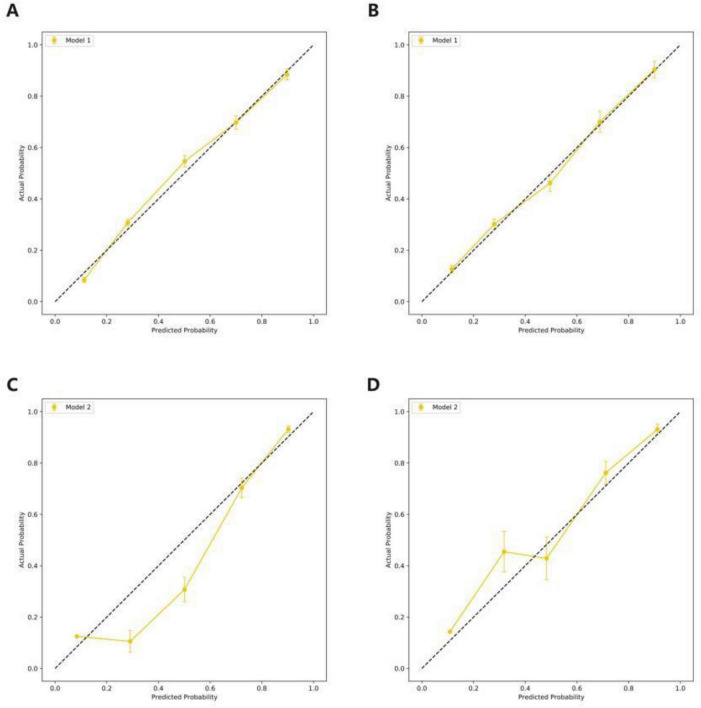
Calibration curves for the training set **(A)** and validation set **(B)** under the SVM machine learning classifier. **(A)** Model 1 training set, **(B)** model 1 validation set, **(C)** model 2 training set, **(D)** model 2 validation set.

Six machine learning classifiers were employed to establish and validate two radiomics models. Model performance was primarily assessed using the validation set area under the AUC. Among the classifiers, the SVM machine learning classifier demonstrated the best performance, with Model 1 achieving an AUC of 0.840. Detailed results for each classifier in Model 1 are presented in Table 4. Model 2 attained an AUC of 0.831, with specific classifier outcomes shown in Table 5. The ROC curves, DCA curves, and calibration curves for the optimal classifier (SVM) in both Model 1 and Model 2 are depicted in Figures 5–7, respectively.

**TABLE 4:** Model results data table for the six machine learning classifiers in model 1.

Classifiers	SVM	RF	SGD	KNN	XGBoost	LightGBM
Training set accuracy	0.788	0.787	0.786	0.745	0.776	0.791
Validation set accuracy	0.776	0.782	0.748	0.751	0.785	0.779
Training set recall index	0.613	0.651	0.651	0.563	0.669	0.669
Verification set recall index	0.581	0.621	0.500	0.589	0.637	0.629
Training set AUC	0.844	0.829	0.828	0.814	0.798	0.818
Validation set AUC	0.840	0.836	0.816	0.792	0.821	0.827
Training set sensitivity	0.613	0.651	0.651	0.563	0.669	0.669
Validation set sensitivity	0.896	0.870	0.868	0.857	0.842	0.866
Training set specificity	0.581	0.621	0.500	0.589	0.637	0.629
Verification set specificity	0.898	0.883	0.904	0.853	0.878	0.873
Training set F1 score	0.688	0.699	0.698	0.627	0.695	0.709
Validation set F1 score	0.667	0.687	0.605	0.646	0.696	0.687

AUC, area under the ROC curve.

**TABLE 5:** Model results data table for the six machine learning classifiers in model 2.

Classifiers	SVM	RF	SGD	KNN	XGBoost	LightGBM
Training set accuracy	0.850	0.857	0.848	0.868	0.850	0.846
Validation set accuracy	0.864	0.818	0.828	0.869	0.793	0.763
Training set recall index	0.959	0.959	0.984	0.981	0.943	0.943
Verification set recall index	0.951	0.883	0.945	0.975	0.834	0.804
Training set AUC	0.844	0.848	0.831	0.792	0.834	0.832
Validation set AUC	0.831	0.782	0.762	0.787	0.793	0.778
Training set sensitivity	0.959	0.959	0.984	0.981	0.943	0.943
Validation set sensitivity	0.407	0.440	0.297	0.407	0.473	0.451
Training set specificity	0.951	0.883	0.945	0.975	0.834	0.804
Verification set specificity	0.457	0.514	0.286	0.371	0.600	0.571
Training set F1 score	0.911	0.915	0.912	0.922	0.910	0.908
Validation set F1 score	0.920	0.889	0.901	0.924	0.869	0.848

AUC, area under the ROC curve.

### Fusion model

Feature-level pre-fusion was performed by combining clinical risk factors and radiomic features selected via LASSO regression. An SVM classifier was then employed to establish the fusion models, exploring whether adding clinical features improved diagnostic performance.

**FIGURE 8:**
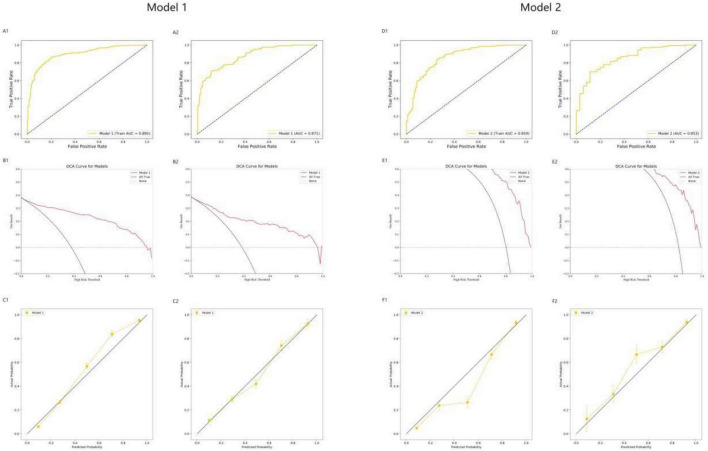
ROC curves **(A,D)**, DCA curves **(B,E)**, and calibration curves **(C,F)** for the fusion model of the training set (1) and validation set (2).

**FIGURE 9:**
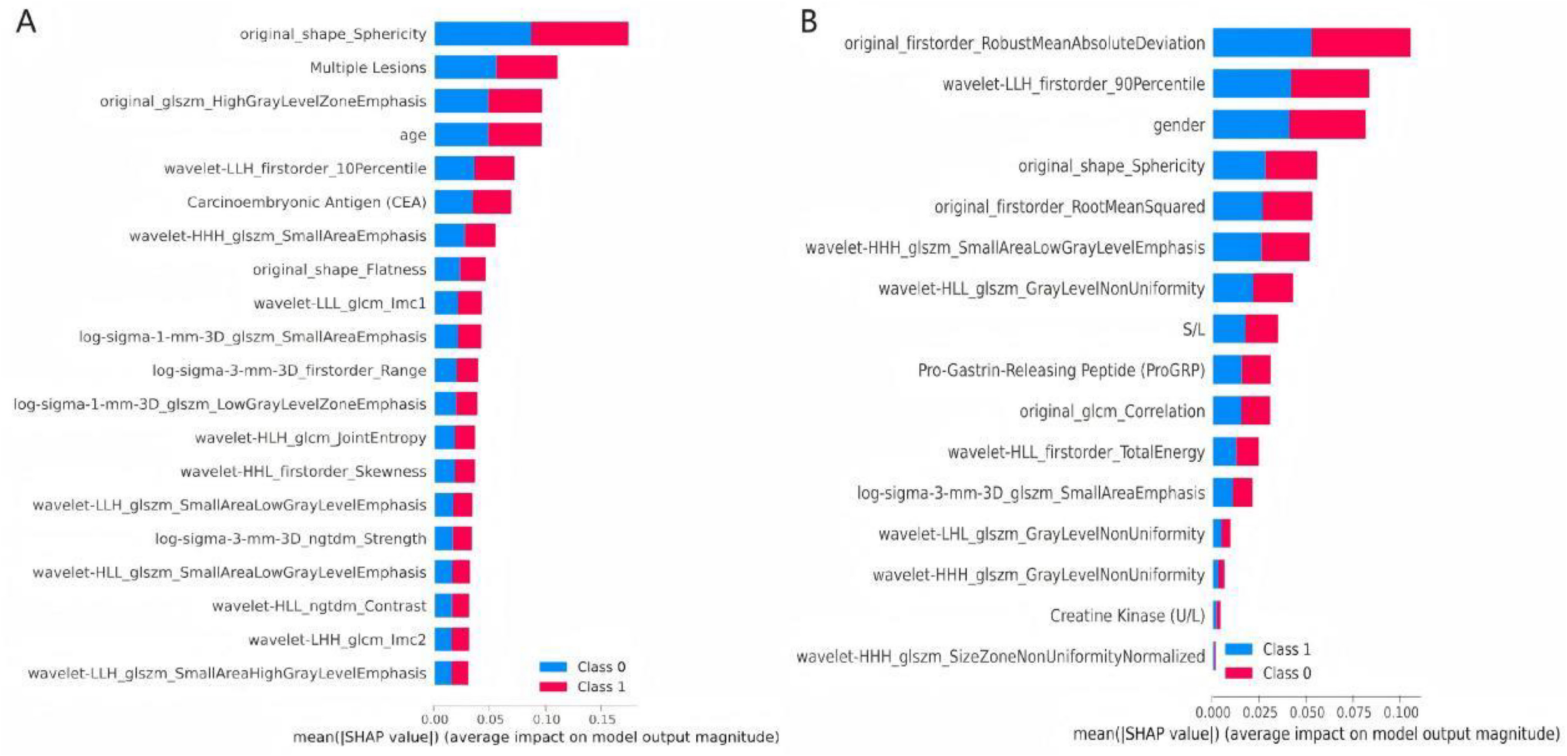
Feature weight SHAP plots for model 1 **(A)** and model 2 **(B)**.

For Model 1, the fusion model achieved a validation set AUC of 0.871, while Model 2 achieved a value of 0.853. DeLong’s test revealed statistically significant differences between models (*p* < 0.05). The ROC curves, DCA curves, and calibration curves for the models are presented in Figure 8. The SHAP weight plots for both models after integrating clinical features are shown in Figure 9.

## Conclusion

In this study, we developed two machine learning models, one for distinguish benign from malignant pulmonary nodules and another for differentiate benign pulmonary nodules from those with atypical/*in situ* carcinoma characteristics. Both models, which integrate clinical risk factors with CT radiomic features, demonstrated strong discriminatory power and offer potential for enhancing non-invasive diagnostic accuracy in clinical practice.

## Discussion

The 2021 WHO Histologic Classification of Lung Tumors (5th ed.) introduced important revisions of lung tumors, including the reclassification of adenocarcinoma *in situ* (AIS) and atypical adenomatoid hyperplasia (AAH) as “glandular precursor lesions,” rather than traditional malignancies (16). These lesions, while generally slow-growing, rarely metastasize, have an excellent prognosis after surgical resection, retain malignant potential that cannot be ignored (17, 18). To this end, we further developed a machine learning model (Model 2) to differentiate benign pulmonary nodules from glandular precursor lesions, demonstrating excellent discriminatory performance.

pGGNs lack specific imaging features (19), as both benign and malignant lesions can present as ground-glass opacities on CT imaging (19–21). Clinicians typically rely on qualitative CT features, such as lesion size, margin characteristics, and solid component ratio, to assess the nature of GGNs. However these methods have limitations in predictive accuracy. For example, patients with pGGN do not exhibit specific clinical manifestations; nearly all ground-glass nodules are detected via CT rather than due to clinical symptoms (22). Future more, for pGGN smaller than 10 mm, the accuracy rate is < 50%. This is primarily due to poor puncture accuracy, low nodule density, and a small number of tumor cells, resulting in insufficient tissue acquisition to support a pathological diagnosis (23). Similarly, pGGNs do not exhibit any serological specificity (24). Blood tumor markers, such as CEA and CYFRA21-1, although having some indicative value, exhibit low sensitivity and specificity in the early stages of lung cancer (25). Thus, misdiagnosis remains a significant issue in clinical practice. And accurate diagnosis is essential for optimizing prognosis, treatment selection, and avoiding unnecessary invasive procedures.

Our study analyzed clinical characteristics (age, BMI, Size, gender, smoking history, tumor history, family history, and location) and test results (blood cell analysis, blood biochemistry, and blood tumor markers) from cases with different pathological type to develop two machine learning models. Model 1 successfully to distinguished benign from malignant pGGNs, while Model 2 further to identified benign nodules with malignant potential. The models demonstrated strong discriminatory power, suggesting that radiomics and deep learning can enhance clinical decision-making by improving diagnostic accuracy.

### Advantages

This study offers several advantages over previous radiomics research. First, unlike prior radiomics studies typically employed a single machine learning method, we used six distinct classifiers (SVM, Random Forest, SGD, KNN, XGBoost, and LightGBM) to construct models. Results revealed significant variations in diagnostic performance among different algorithms within the validation set. Relying on a single algorithm may compromise model accuracy due to the inherent limitations of the chosen method. Therefore, integrating multiple algorithms enhances the predictive accuracy and reliability of the model.

Second, the widespread use of chest CT in routine medical practice has led to an increased detection rate of pGGNs. These cases present diagnostic challenges. Our models help address this challenge by incorporating clinical and radiomic data, reducing the risk of unnecessary invasive procedures while ensuring accurate diagnoses. Furthermore, we also considered the special group of glandular precursor lesions with malignant potential. Different pathological types necessitate individualized treatment planning, thereby avoiding both over-testing and missed diagnoses. Given the scarcity of studies focusing on the differential diagnosis of specific pathological types, the innovation of this research is evident.

Third, our comprehensive analysis combined clinical and imaging characteristics with radiomic features. By conducting univariate and multivariate logistic regression analyses, we identified several significant features that enhanced diagnostic performance, offering valuable support for clinical interpretation.

Finally, integrating deep learning tools into our model further improved its performance. This technological enhancement allows for more sophisticated and objective analysis, contributing to higher model accuracy and reliability.

### Limitations

Despite its strengths, this study has several limitations. The retrospective nature may introduce bias in the collection of clinical characteristics, and prospective studies would allow for more comprehensive model validation. Additionally, the single-center design of this study may limit its generalizability. Finally, the sample size of certain pathological subtypes was limited, which may have affected the robustness of the model for specific subtypes. To address these limitations, we plan to extend the study duration and further promote multicenter collaboration, while conducting more detailed and in-depth analyses of pathological subtypes.

## Data Availability

The raw data supporting the conclusions of this article will be made available by the authors, without undue reservation.
