# Supplementary material for: Development of a CT-based comprehensive model with deep learning for differentiating pathological types of pulmonary ground-glass nodules
Source: Front Med (Lausanne). 2026 May 26;13:1831127. doi: 10.3389/fmed.2026.1831127 (PMC13247685; doi:10.3389/fmed.2026.1831127)
Supplement: Supplementary file 1 [file Table_1.docx]

Appendix table I Single factor analysis of Model I

| **Variable** | **OR** | **CI95_lower** | **CI95_upper** | **P_value** |
| --- | --- | --- | --- | --- |
| Age | 1.038601825 | 1.023414945 | 1.054014069 | 4.66643E-07 |
| Carcinoembryonic_Antigen__CEA_ | 1.337603973 | 1.179393288 | 1.517037961 | 5.92532E-06 |
| Cytokeratin | 1.196024458 | 1.017317897 | 1.406123404 | 0.030165875 |
| Multiple_Lesions | 0.731288408 | 0.543458509 | 0.984035997 | 0.038808116 |
| Amylase | 0.992734683 | 0.985620396 | 0.999900321 | 0.046908077 |
| Platelet_Distribution_Width__fL_ | 1.063986302 | 0.999680539 | 1.132428618 | 0.051185787 |
| Albumin_Globulin_Ratio | 0.585964764 | 0.341378475 | 1.005788968 | 0.052498473 |
| Total_Cholesterol__mmol_L_ | 0.849042373 | 0.719269581 | 1.002229165 | 0.053155764 |
| Small_Dense_LDL_Cholesterol | 0.629489162 | 0.387341765 | 1.023015438 | 0.061744945 |
| Glutathione_Reductase | 0.984430556 | 0.968123415 | 1.001012375 | 0.065587062 |
| Chloride__mmol_L_ | 1.064767203 | 0.991130975 | 1.143874246 | 0.086103035 |
| Triglycerides__mmol_L_ | 0.851070152 | 0.707203176 | 1.024204116 | 0.087853356 |
| Albumin__g_L_ | 0.966324591 | 0.927408978 | 1.006873168 | 0.102393179 |
| Irregular_Edge | 1.286016794 | 0.950050064 | 1.740791626 | 0.103464854 |
| Sialic_Acid__mg_L_ | 1.002040975 | 0.999549836 | 1.004538322 | 0.108400218 |
| C(Location, Treatment)[T.3] | 0.733333333 | 0.492697546 | 1.091496765 | 0.126388064 |
| Retraction_Present | 1.301163393 | 0.919564075 | 1.841118223 | 0.137152801 |
| Pro_Gastrin_Releasing_Peptide__ProGRP_ | 1.008022396 | 0.997337738 | 1.018821521 | 0.141656856 |
| family_history | 1.666666667 | 0.83665363 | 3.320104852 | 0.146290345 |
| anti_inflammatory_therapy | 2.471223022 | 0.69129834 | 8.834019795 | 0.163936505 |
| Platelet_Count | 0.998007303 | 0.995198275 | 1.00082426 | 0.165430456 |
| LDL_Cholesterol | 0.8861698 | 0.734744117 | 1.06880327 | 0.206232102 |
| Sodium__mmol_L_ | 1.047235985 | 0.970829103 | 1.129656296 | 0.23245542 |
| diabetes | 1.356671632 | 0.817941822 | 2.250230845 | 0.237389068 |
| Hypertension | 1.211538462 | 0.84213511 | 1.742980937 | 0.301100631 |
| S_L | 1.218614955 | 0.837386623 | 1.77340116 | 0.301667853 |
| gender | 1.178756477 | 0.855337894 | 1.62446542 | 0.314876671 |
| Globulin__g_L_ | 1.01863167 | 0.980966709 | 1.057742806 | 0.336901936 |
| Urea__mmol_L_ | 0.943665558 | 0.837723637 | 1.063005324 | 0.339914635 |
| Neuron_Specific_Enolase__NSE_ | 0.980092846 | 0.940255564 | 1.021617977 | 0.342233682 |
| Direct_Bilirubin__μmol_L_ | 0.959778218 | 0.878220279 | 1.048910223 | 0.364903958 |
| HDL_Cholesterol | 0.810280251 | 0.494025899 | 1.328987176 | 0.404655892 |
| Lactate_Dehydrogenase | 0.998199241 | 0.993856002 | 1.00256146 | 0.417868335 |
| Prealbumin__g_L_ | 0.329393784 | 0.022010877 | 4.929393095 | 0.421152411 |
| α_Hydroxybutyrate_Dehydrogenase__U_L_ | 0.997649798 | 0.991807521 | 1.003526489 | 0.432330886 |
| _Total_Bilirubin__μmol_L_ | 0.988383369 | 0.959593356 | 1.018037149 | 0.438506591 |
| BMI | 1.006372156 | 0.990219977 | 1.022787804 | 0.441633765 |
| Neutrophil_Count__10_9_L_ | 0.958581139 | 0.859979641 | 1.068487853 | 0.444978088 |
| White_Blood_Cell_Count__10_9_L_ | 0.963928716 | 0.876758838 | 1.059765274 | 0.447455536 |
| Size_mm_ | 0.991872879 | 0.970524912 | 1.013690422 | 0.462286226 |
| Glycocholic_Acid__mg_L_ | 0.963866651 | 0.873588413 | 1.063474408 | 0.463277173 |
| Gamma_Glutamyl_Transferase__U_L_ | 0.99814582 | 0.992664623 | 1.003657283 | 0.508880809 |
| Calcium__mmol_L_ | 0.639222733 | 0.166587851 | 2.45279413 | 0.514245576 |
| Uric_Acid__μmol_L_ | 0.999296943 | 0.997167672 | 1.001430762 | 0.518126268 |
| Hemoglobin__g_L_ | 0.996430375 | 0.985629587 | 1.007349521 | 0.520163552 |
| Indirect_Bilirubin__μmol_L_ | 0.987210276 | 0.948803452 | 1.027171779 | 0.524912558 |
| Total_Bile_Acids | 0.988846513 | 0.954009271 | 1.024955895 | 0.539921303 |
| Iron__μmol_L_ | 1.008690441 | 0.98114641 | 1.037007723 | 0.540173658 |
| tumor_history | 1.144540943 | 0.727419541 | 1.800850673 | 0.559367216 |
| Aspartate_Aminotransferase__U_L_ | 1.003821186 | 0.990977946 | 1.016830878 | 0.561573695 |
| smoking_history | 1.146285714 | 0.720159073 | 1.824556529 | 0.564822834 |
| Red_Blood_Cell_Count__10_12_L_ | 0.905560977 | 0.643621731 | 1.274103472 | 0.569061199 |
| Alanine_Aminotransferase__U_L_ | 0.998412185 | 0.992225779 | 1.004637163 | 0.616307967 |
| Creatine_Kinase__U_L_ | 1.000820586 | 0.997585882 | 1.004065779 | 0.619465925 |
| Creatine_Kinase_MB__ng_mL_ | 1.025137785 | 0.915163908 | 1.148327058 | 0.668067288 |
| Magnesium__mmol_L_ | 1.642402217 | 0.16878994 | 15.98131405 | 0.669085004 |
| Total_Protein__g_L_ | 0.994859901 | 0.968824891 | 1.021594545 | 0.703286749 |
| C(Location, Treatment)[T.5] | 1.094527363 | 0.684514658 | 1.750130745 | 0.706051005 |
| Creatinine__μmol_L_ | 0.997674589 | 0.985371954 | 1.010130827 | 0.713059854 |
| Alkaline_Phosphatase__U_L_ | 0.998650796 | 0.991462735 | 1.00589097 | 0.714131035 |
| C(Location, Treatment)[T.4] | 0.88 | 0.406018973 | 1.907300034 | 0.746008828 |
| C(Location, Treatment)[T.2] | 0.935873016 | 0.612250969 | 1.430554374 | 0.759514449 |
| Hematocrit | 0.993888428 | 0.95523791 | 1.03410281 | 0.761950135 |
| Cholinesterase | 0.999987153 | 0.999902049 | 1.000072264 | 0.767340142 |
| Lymphocyte_Count__10_9_L_ | 0.955894664 | 0.707354671 | 1.291763022 | 0.769059343 |
| Potassium__mmol_L_ | 1.064333441 | 0.651714412 | 1.738193374 | 0.803254545 |
| Phosphorus__mmol_L_ | 0.909224729 | 0.343412999 | 2.407275235 | 0.848085259 |
| β2_Microglobulin__mg_L_ | 1.02144253 | 0.775865668 | 1.344749337 | 0.879807638 |
| Lipase | 0.999370132 | 0.991154294 | 1.007654072 | 0.881083948 |
| Homocysteine | 0.997808594 | 0.968860948 | 1.027621138 | 0.883881253 |
| Free_Fatty_Acids | 0.958993567 | 0.506402541 | 1.81608224 | 0.897739064 |
| Eosinophil_Count | 1.080808865 | 0.325238421 | 3.591666079 | 0.899076754 |
| Basophil_Count | 0.719095845 | 2.9263E-05 | 17670.74569 | 0.949024128 |
| Adenosine_Deaminase__U_L_ | 1.001227157 | 0.957294397 | 1.047176108 | 0.957278019 |
| Monocyte_Count__10_9_L_ | 0.991387223 | 0.297311127 | 3.305791598 | 0.988768185 |

Appendix table II Single factor analysis of Model II

| **Variable** | **OR** | **CI95_lower** | **CI95_upper** | **P_value** |
| --- | --- | --- | --- | --- |
| Pro_Gastrin_Releasing_Peptide__ProGRP_ | 0.966947995 | 0.949656069 | 0.984554783 | 0.000261569 |
| gender | 0.433306531 | 0.269872788 | 0.695715012 | 0.000536604 |
| Globulin__g_L_ | 0.932809032 | 0.881332393 | 0.987292306 | 0.01632632 |
| S_L | 0.525413989 | 0.298891496 | 0.923612294 | 0.025348188 |
| Eosinophil_Count | 0.111617555 | 0.015313565 | 0.81355832 | 0.030497994 |
| Albumin__g_L_ | 1.070226128 | 1.005162438 | 1.13950136 | 0.03393273 |
| smoking_history | 0.496142433 | 0.256655609 | 0.95909579 | 0.037146235 |
| Creatine_Kinase_MB__ng_mL_ | 0.842939539 | 0.71691584 | 0.991116427 | 0.038643346 |
| C(Location, Treatment)[T.3] | 0.520540392 | 0.273867417 | 0.989392247 | 0.046315119 |
| Glycocholic_Acid__mg_L_ | 0.893704972 | 0.796885905 | 1.002287243 | 0.054742727 |
| C(Location, Treatment)[T.4] | 0.41781874 | 0.149509279 | 1.167636556 | 0.096034572 |
| Phosphorus__mmol_L_ | 0.286458321 | 0.063508766 | 1.292079426 | 0.103830808 |
| Neuron_Specific_Enolase__NSE_ | 0.959021133 | 0.910808908 | 1.009785394 | 0.111848137 |
| Chloride__mmol_L_ | 1.085596844 | 0.97934186 | 1.203380101 | 0.118107931 |
| Cholinesterase | 0.999901561 | 0.999775995 | 1.000027143 | 0.124447704 |
| Hypertension | 0.65597148 | 0.378338246 | 1.137338311 | 0.133190265 |
| Lymphocyte_Count__10_9_L_ | 0.729420771 | 0.478667687 | 1.11153244 | 0.142109981 |
| Magnesium__mmol_L_ | 0.075221741 | 0.002316758 | 2.442339543 | 0.145093478 |
| Carcinoembryonic_Antigen__CEA_ | 0.863415398 | 0.70387676 | 1.059114594 | 0.158848569 |
| LDL_Cholesterol | 1.228309159 | 0.915123769 | 1.64867687 | 0.170892869 |
| Cytokeratin | 0.846521362 | 0.665971234 | 1.076020074 | 0.173408304 |
| Adenosine_Deaminase__U_L_ | 0.956504805 | 0.894956612 | 1.022285807 | 0.190044814 |
| Prealbumin__g_L_ | 15.15915411 | 0.204958561 | 1121.202024 | 0.215666985 |
| Size_mm_ | 1.024310258 | 0.985715551 | 1.064416103 | 0.2202927 |
| diabetes | 0.6039065 | 0.269373521 | 1.353893507 | 0.220802736 |
| Platelet_Count | 1.002745159 | 0.998287639 | 1.007222584 | 0.227814183 |
| Gamma_Glutamyl_Transferase__U_L_ | 0.998830013 | 0.996898595 | 1.000765173 | 0.235844916 |
| Sialic_Acid__mg_L_ | 1.002350942 | 0.998294818 | 1.006423546 | 0.256361672 |
| White_Blood_Cell_Count__10_9_L_ | 0.924367117 | 0.805607513 | 1.060633811 | 0.26231384 |
| Basophil_Count | 0.000231391 | 8.57636E-11 | 624.2959882 | 0.267851556 |
| C(Location, Treatment)[T.5] | 0.653763441 | 0.300365427 | 1.422955501 | 0.284147454 |
| Lipase | 1.008513417 | 0.991912691 | 1.025391973 | 0.316792002 |
| Amylase | 1.005486151 | 0.994714574 | 1.016374371 | 0.319441773 |
| Irregular_Edge | 1.275857081 | 0.788739294 | 2.063814117 | 0.320799337 |
| Direct_Bilirubin__μmol_L_ | 0.954197371 | 0.86659312 | 1.050657573 | 0.339971804 |
| Hemoglobin__g_L_ | 0.991624872 | 0.974613631 | 1.008933034 | 0.340777968 |
| Calcium__mmol_L_ | 0.390122113 | 0.054584514 | 2.788249853 | 0.348210012 |
| Homocysteine | 0.977454582 | 0.930226249 | 1.027080735 | 0.366806961 |
| Hematocrit | 0.972804573 | 0.914685388 | 1.034616655 | 0.380359781 |
| Sodium__mmol_L_ | 0.952311163 | 0.850585034 | 1.066203278 | 0.396565172 |
| Creatinine__μmol_L_ | 1.008107799 | 0.988583681 | 1.02801751 | 0.418361287 |
| Free_Fatty_Acids | 1.474517764 | 0.561996913 | 3.868709214 | 0.430079698 |
| age | 0.992656153 | 0.974310754 | 1.01134698 | 0.438659238 |
| Triglycerides__mmol_L_ | 0.905316549 | 0.703434234 | 1.165138138 | 0.439703038 |
| Iron__μmol_L_ | 1.014481417 | 0.97435156 | 1.056264071 | 0.48505688 |
| Multiple_Lesions | 1.175921121 | 0.74273513 | 1.861754515 | 0.489396564 |
| Glutathione_Reductase | 0.991142947 | 0.966407846 | 1.01651114 | 0.490229236 |
| Small_Dense_LDL_Cholesterol | 1.277009936 | 0.614652999 | 2.653130108 | 0.512199441 |
| Monocyte_Count__10_9_L_ | 0.549928582 | 0.08832803 | 3.423844554 | 0.521601917 |
| Total_Cholesterol__mmol_L_ | 1.078413915 | 0.839434524 | 1.385428569 | 0.554776888 |
| Urea__mmol_L_ | 1.051518151 | 0.872520017 | 1.267237884 | 0.597754195 |
| Neutrophil_Count__10_9_L_ | 0.958823873 | 0.817883351 | 1.124051759 | 0.604209428 |
| family_history | 1.491620112 | 0.327922389 | 6.784930311 | 0.604905539 |
| Potassium__mmol_L_ | 0.817446984 | 0.375055038 | 1.781657367 | 0.612102582 |
| BMI | 0.991688312 | 0.959999967 | 1.024422648 | 0.614456534 |
| _Total_Bilirubin__μmol_L_ | 0.991277607 | 0.952169568 | 1.031991913 | 0.669683635 |
| Total_Bile_Acids | 0.986816834 | 0.927835392 | 1.04954766 | 0.67299595 |
| Red_Blood_Cell_Count__10_12_L_ | 0.897543406 | 0.522000961 | 1.543261844 | 0.695877627 |
| Lactate_Dehydrogenase | 0.998783562 | 0.992616218 | 1.004989225 | 0.700125403 |
| α_Hydroxybutyrate_Dehydrogenase__U_L_ | 0.998419902 | 0.990267367 | 1.006639554 | 0.705414428 |
| Total_Protein__g_L_ | 0.992538164 | 0.953167717 | 1.033534802 | 0.716835768 |
| Creatine_Kinase__U_L_ | 0.999232601 | 0.99486004 | 1.003624379 | 0.731526587 |
| Alkaline_Phosphatase__U_L_ | 1.001415032 | 0.992582496 | 1.010326165 | 0.754406954 |
| Aspartate_Aminotransferase__U_L_ | 0.997693824 | 0.983110374 | 1.012493605 | 0.758602867 |
| Platelet_Distribution_Width__fL_ | 0.98651131 | 0.894764313 | 1.087665826 | 0.785101089 |
| tumor_history | 0.911538462 | 0.460604958 | 1.803937088 | 0.790278658 |
| Retraction_Present | 1.064220183 | 0.646691667 | 1.751320848 | 0.806532082 |
| Uric_Acid__μmol_L_ | 1.000362505 | 0.997088029 | 1.003647734 | 0.828470231 |
| Alanine_Aminotransferase__U_L_ | 1.000388402 | 0.993839588 | 1.006980368 | 0.907744027 |
| HDL_Cholesterol | 0.959131449 | 0.46362989 | 1.984197215 | 0.910423943 |
| C(Location, Treatment)[T.2] | 1.044546851 | 0.481116268 | 2.267805509 | 0.912259838 |
| Indirect_Bilirubin__μmol_L_ | 0.997993565 | 0.942689173 | 1.056542478 | 0.944950711 |
| anti_inflammatory_therapy | 0.98265896 | 0.389449898 | 2.479442506 | 0.970449285 |
| β2_Microglobulin__mg_L_ | 0.994314745 | 0.685531757 | 1.442182366 | 0.976026471 |
